# Research on the impact mechanism of Green Emotional Involvement on Consumer Engagement Behavior: the chain mediating roles of Perceived Authenticity and Perceived Value

**DOI:** 10.3389/fpsyg.2025.1687837

**Published:** 2026-01-15

**Authors:** Yike Yang, Qiaolan Su

**Affiliations:** 1 School of Food Science and Engineering, Hainan University, Haikou, China; 2 School of Tourism and Sports Health, Hezhou University, Hezhou, China

**Keywords:** Green Emotional Involvement, Consumer Engagement Behavior, Theory of Planned Behavior, Perceived Value, Perceived Authenticity

## Abstract

Throughout the 15th Five-Year Plan, China will enter a decisive phase of structural realignment and rapid growth-model transformation. At the nexus of the “dual-carbon” targets and high-quality development, green consumption—propelled by the “Two New” policy package—is set to scale up faster. Therefore, understanding how emotional factors shape consumers’ engagement with green products has become increasingly important. This study aims to explore how Green Emotional Involvement (GEI) influences Consumer Engagement Behavior (CEB), and to examine the mediating effects of Perceived Value (PV) and Perceived Authenticity (PA), as well as the moderating role of gender. Based on the Theory of Planned Behavior (TPB) and Involvement Theory, this study employed a questionnaire survey of 274 Chinese consumers. CB-SEM excels at disentangling the subtle, multilayered interplay among a multitude of variables. Therefore the data were analyzed using Structural Equation Modeling (SEM) and Multi-group Analysis (MGA) to test the hypothesized relationships. The results show that GEI has a significant positive effect on CEB. PV mediates this relationship significantly, while the mediating effect of PA is not supported. Furthermore, gender moderates the relationship between GEI and PA, indicating that female consumers are more sensitive to authenticity cues. The study provides new empirical evidence that consumers’ emotional involvement can foster engagement through Perceived Value enhancement, while authenticity perceptions play a more limited role. The findings enrich the TPB framework by integrating emotional and cognitive factors and offer practical guidance for developing gender-differentiated green marketing strategies aimed at enhancing consumer engagement and loyalty.

## 1 Introduction

With the escalating severity of global environmental issues, green food consumption has gradually become an important development trend in the consumption market (Chu et al., 2023; Li M. et al., 2024). An increasing number of consumers are paying attention to the green attributes of food product—such as being organic, pollution-free, and secure—to meet their needs for health and environmental protection (Deng et al., 2024). According to the *China Green Food Development Statistical Yearbook* released by National Bureau of Statistics of China, in 2024 the domestic sales of green food in China reached 609.78 billion RMB, showing a significant increase compared to 2018. However, alongside the rapid growth of green food consumption, new phenomena and changes have emerged. On one hand, the green food market is becoming more diversified and segmented, with existing green food varieties meeting the heterogeneous demands of consumer groups (Jaiswal et al., 2021; Rousseau and Vranken, 2013). In January 2025, the *Opinions on Further Deepening Rural Reform and Solidly Promoting Comprehensive Rural Revitalization* issued by the Central Committee of the Communist Party of China and the State Council proponent of forest-based food products to enrich the forest grain reserve, improve the quality and efficiency of the edible mushroom industry, promote the development of algae-based foods, and foster bio-agriculture to explore new food resources. On the other hand, consumers’ cognition and evaluation criteria for green food are continuously rising (Barbarossa and Pastore, 2015; Kamboj and Kishor, 2022). Rather than relying solely on green labels, they now place greater emphasis on the green values and environmental significance embodied behind the products (Chakraborty et al., 2022). This raises a critical question: what factors influence consumers’ Consumer Engagement Behavior toward green food? Is it primarily their personal attention to green food is Green Emotional Involvement or are there other influential factors at play? Therefore, an in-depth investigation into the influence of Green Emotional Involvement on consumers’ engagement with green food and its underlying mechanisms holds significant value for promoting the development of the green food market and guiding consumers toward forming a sustainable green consumption mindset.

In the field of green consumption, numerous scholars have conducted extensive research on the factors influencing green food consumption. Early studies primarily focused on consumers’ general green consumption behaviors, such as their purchase intentions, attitudes, and behavioral intentions toward green products. For example, Horlings (2015) from the perspective of a multi-group structural equation model, analyzed the influencing factors of green purchasing behavior and found that consumers’ environmental awareness and personal values had a significant impact on their green purchasing behaviors. Similarly, Shin and Hancer (2016) based on the Theory of Planned Behavior, examined the purchase behavior of environmentally friendly products and pointed out that factors such as subjective norms and perceived behavioral control play important roles in consumers’ purchase decisions. In recent years, the *China Agricultural* Green Development Report 2022 indicated that by the end of 2022, there were 27,246 certified entities for green food and organic agricultural products nationwide, with a total of 60,254 products—representing year-on-year increases of 10% and 8.3%, respectively. The supply of high-quality green agricultural products continues to grow. This suggests that the development of the green food market is inseparable from the improvement of consumers’ cognition and acceptance of green food (Iglesias et al., 2019). As an important indicator of consumers’ level of attention to green food, Green Emotional Involvement has gradually attracted increasing attention from scholars.

Green Emotional Involvement reflects consumers’ interest in green food, the degree of importance they attach to it, as well as the effort and emotions they invest (Jang et al., 2015). Relevant studies indicate that consumers with high Green Emotional Involvement are more easily attracted by the green attributes of green food, hold more positive perceptions and evaluations of such products, and are therefore more likely to engage in behaviors aligned with green food (Woo and Kim, 2019). For example, Mathew et al. (2014) found that compared with low-involvement consumers, high-involvement consumers pay greater attention to green advertisements or green products and hold more positive attitudes toward advertisements, providing preliminary theoretical support for studying the influence of Green Emotional Involvement on Consumer Engagement Behavior. In recent years, many scholars have conducted in-depth explorations of the relationship between Green Emotional Involvement and consumer behavior, achieving fruitful results. For instance, Sun et al. (2019) reviewed residents’ green consumption behaviors and pointed out that consumers’ Green Emotional Involvement is an important factor influencing green consumption behavior. Sheng et al. (2019) examined the mechanism through which consumer values under the Chinese cultural context affect green consumption intention, finding that Green Emotional Involvement plays a key role. Ali et al. (2023) assessed the impact of green consumption behavior and purchase intention on environmental sustainability, emphasizing the positive significance of Green Emotional Involvement in promoting green consumption behavior. In addition, Sun et al. (2019) studied the factors influencing public green consumption behavior under the carbon peaking goal and found that Green Emotional Involvement has a significant positive effect on consumers’ green consumption behavior. Zhang et al. (2019) measured the green consumption development index of Shandong Province, further confirming the importance of Green Emotional Involvement in green consumption behavior.

In summary, existing studies exploring the factors influencing green food consumption and the relationship between Green Emotional Involvement and consumer behavior have achieved certain results, providing a solid theoretical foundation for this research. Therefore, an in-depth investigation into how Green Emotional Involvement (GEI) shapes consumers’ engagement with green food and the underlying psychological mechanisms is essential for both theory and practice. Although prior studies have explored green purchase intention, few have examined how emotional involvement leads to multidimensional engagement behaviors such as interaction, recommendation, and participation. Addressing this gap can enrich our understanding of consumers’ sustained engagement in green markets. This study advances the Theory of Planned Behavior (TPB) by incorporating emotional and cognitive pathways to explain engagement behavior. By integrating Perceived Authenticity (PA) and Perceived Value (PV) as mediators, it captures both affective resonance and rational evaluation processes, offering a more holistic model of green consumer engagement. The study innovatively introduces a dual-mediation and gender-moderation framework, revealing how gender differences alter authenticity perception within green emotional marketing contexts. These insights provide fresh empirical evidence for understanding emotional mechanisms in sustainable consumption and strategic guidance for green marketing communication.

## 2 Theoretical basis and research hypotheses

### 2.1 Theory of planned behavior

The Theory of Planned Behavior (TPB) was proposed by Ajzen in 1991. This theory posits that an individual’s behavioral intention is the key factor in predicting their actual behavior, and behavioral intention is influenced by three main factors: attitude (Emekci, 2019), subjective norm (Godin and Kok, 1996), and perceived behavioral control (Yuriev et al., 2020). Attitude refers to an individual’s evaluation and preference toward a specific behavior (Ajzen and Fishbein, 2000); subjective norm refers to the perceived social pressure—namely, the expectations of people around them regarding their behavior (Rimal and Real, 2005); and perceived behavioral control refers to an individual’s perceived ease or difficulty in performing the specific behavior (Ajzen, 2002).

In this study, the Theory of Planned Behavior (TPB) serves as the theoretical foundation for constructing the research model on the influence of Green Emotional Involvement on Consumer Engagement Behavior. TPB has been widely applied in fields such as consumer behavior (Emekci, 2019), health behavior (Godin and Kok, 1996), and environmental behavior (Yuriev et al., 2020), offering a comprehensive perspective for examining individual behavior. It effectively explains the relationship between behavioral intention and actual behavior, as well as how individual attitudes, social pressure, and perceived control jointly shape behavioral intentions and subsequently influence actual behavior (Ajzen, 2002). Green Emotional Involvement, as an indicator of consumers’ attention to green food, can be viewed as analogous to the attitude component in TPB (Qi and Ploeger, 2019). It reflects consumers’ positive or negative evaluations of green food and their perception of its importance (Aschemann-Witzel and Zielke, 2017). Consumers with high Green Emotional Involvement tend to hold more positive attitudes toward green food and are therefore more likely to develop an intention to engage in such behavior (Iqbal et al., 2021). Perceived Value is related to the perceived behavioral control component in TPB (Zolait, 2014). The higher the Perceived Value of green food, the fewer obstacles consumers perceive in purchasing and consuming it, leading to stronger perceived behavioral control and a greater likelihood of transforming behavioral intention into actual action (Lim et al., 2014). Perceived Authenticity can be considered a factor that reinforces both attitudes and subjective norms (Girish and Lee, 2020). When consumers perceive higher authenticity in green food, their positive attitudes become more stable, and they experience stronger positive social pressure from their environment, which in turn increases the likelihood of engaging in Consumer Engagement Behavior (Vermeir and Verbeke, 2006). Consistent with the Theory of Planned Behavior (TPB), this study posits that Green Emotional Involvement impacts Consumer Engagement Behavior via its effects on Perceived Value and Perceived Authenticity. This process aligns with the behavioral mechanism described by TPB and provides a sound theoretical rationale for this study.

### 2.2 Research hypotheses

#### 2.2.1 The influence of Green Emotional Involvement on Consumer Engagement Behavior

Since its introduction by Sherif and Cantril (1947), involvement theory has gradually evolved along two classic lines of research: the first emphasizes the moderating effects of the direction, intensity, and manifestation of involvement on purchase intention and behavior (Drossos et al., 2014); the second highlights individuals’ attitudinal and behavioral responses arising from subjective associations between their interests, goals, or needs and a given object (Van Tonder et al., 2023). Zaichkowsky (1994) further classified involvement into advertising involvement, product involvement, and purchase decision involvement based on the nature of the involvement object; Wirth (2013), focusing on the essence of involvement, distinguished between cognitive involvement and emotional involvement.

Extending these frameworks to the green consumption context, consumers’ level of attention to the environmental attributes and health value of products is defined as green involvement, which serves as a crucial psychological nexus linking green cognition with sustained behavioral performance. At present, there is no universally accepted definition of Green Emotional Involvement. Based on existing research, it can be interpreted as the degree to which individuals pay attention to information related to a product’s environmental attributes and health value, as well as the resulting tendency toward certain consumption behaviors (Mancini et al., 2017). Such a tendency can directly or indirectly influence consumers’ decision-making processes when purchasing green products, including information search, product evaluation, and final purchase (Maniatis, 2016).

In the green consumption field, Van Tonder et al. (2020), in studying the mechanism through which green cognition affects consumers’ green purchasing behavior, constructed a structural equation model incorporating Green Emotional Involvement as a mediating variable. Zhang (2025), in examining the impact of benefit perception on consumers’ green brand attachment, argued that Green Emotional Involvement positively influences green brand attachment.

Based on the above analysis, it can be seen that variables related to Green Emotional Involvement can influence Consumer Engagement Behavior in the green consumption context. Therefore, this study, drawing on the concept of Green Emotional Involvement within the context of green food and building upon the literature review above, proposes the following hypothesis:

H1: Green Emotional Involvement has a significant positive effect on Consumer Engagement Behavior.

#### 2.2.2 The mediating role of Perceived Value

Perceived Value is defined as the consumer’s subjective assessment of the overall utility of a green product after weighing the benefits obtained against the costs incurred (Zeithaml, 1988). In the context of green consumption, Perceived Value encompasses not only functional benefits (e.g., quality, health) and emotional benefits (e.g., moral satisfaction, self-expression), but also altruistic value stemming from contributions to environmental protection (Joshi et al., 2021). Existing research generally categorizes the factors influencing into three dimensions: Individual factors, such as Green Emotional Involvement, environmental knowledge, and self-congruence (Li J. et al., 2024); Product factors, such as green attributes, certification labels, and brand reputation (Maniatis, 2016); and situational factors, such as social norms, credibility of information, and information availability (Gupta et al., 2024). In terms of behavioral outcomes, high Perceived Value has been shown to significantly enhance consumer satisfaction, repurchase intention, word-of-mouth recommendation, and the depth of long-term brand relationships (Manzoor et al., 2022).

The enhancing effect of Green Emotional Involvement on Perceived Value is reflected in two main pathways. First, consumers with high Green Emotional Involvement tend to process green-related information more elaborately, thereby better identifying the multiple benefits of green products in terms of health, environmental protection, and social recognition, which in turn improves their overall utility judgments (Maniatis, 2016). Second, the higher the emotional involvement, the more likely consumers are to regard green consumption as an important form of self-identity expression, further amplifying the symbolic and emotional value of green products and ultimately elevating Perceived Value (Hasudungan and Saragih, 2024).

Once Perceived Value is enhanced, Consumer Engagement Behavior is also strengthened. High Perceived Value not only indicates that consumers hold positive evaluations of the functional and emotional utility of green products but also reflects higher trust and identification with the brand (Khan and Mohsin, 2017). This motivates them to invest more time, effort, and emotional resources in interactions, co-creation, and word-of-mouth communication, thus fostering sustained and deeper brand engagement (Hsieh and Chang, 2016). Therefore, Green Emotional Involvement indirectly promotes Consumer Engagement Behavior by enhancing Perceived Value.

H2: Perceived Value plays a significant mediating role between Green Emotional Involvement and Consumer Engagement Behavior.

#### 2.2.3 The mediating role of Perceived Authenticity

Perceived Authenticity refers to consumers’ subjective judgment regarding the truthfulness, reliability, and consistency of the environmental attributes, sustainability commitments, and underlying value propositions claimed by a green product or brand (Olk, 2021). In the context of green consumption, Perceived Authenticity involves not only the verifiability of product functionality and environmental performance, but also whether cues such as brand narratives, certification labels, and supply chain transparency align with consumers’ expectations and ethical standards (Nguyen et al., 2025).

Previous studies have summarized the factors influencing Perceived Authenticity into three main categories. First, at the individual level, Green Emotional Involvement, environmental knowledge, and skepticism determine consumers’ sensitivity to authenticity cues and the depth of their information processing (Goh and Balaji, 2016). Second, at the information level, the argument quality of green advertising, third-party certifications, and traceability systems significantly enhance perceptions of authenticity (Marschlich and Hurtado, 2025). Third, at the brand level, corporate reputation, historical consistency, and corporate social responsibility (CSR) communication strategies strengthen Perceived Authenticity by enhancing trust and identification (Blombäck and Scandelius, 2013). In terms of behavioral outcomes, high Perceived Authenticity has been shown to reduce perceived risk, increase brand trust, promote word-of-mouth recommendations, and deepen consumer–brand relationships (Liang et al., 2018).

Green Emotional Involvement has a significant positive effect on Perceived Authenticity (Pittman et al., 2022). Consumers with high Green Emotional Involvement are more inclined to invest cognitive resources in searching for, comparing, and verifying green information, paying close attention to critical cues such as certification labels, raw material sources, and production processes. This enables them to more readily identify and accept green claims endorsed by authoritative sources, thereby reducing uncertainty caused by misinformation (Wang et al., 2018). In addition, high involvement strengthens individuals’ self-congruence with green values, encouraging them to adopt the brand’s sustainability narrative with lower defensive motivation, thereby improving their authenticity evaluations of the brand’s green commitments (Japutra et al., 2023).

When Perceived Authenticity is strengthened, Consumer Engagement Behavior is enhanced accordingly. Authenticity not only reduces consumers’ search and decision-making costs but also fosters trust and emotional identification, motivating them to invest time, effort, and emotional resources in ongoing interactions, value co-creation, and positive word-of-mouth communication (Hollebeek and Macky, 2019).

Therefore, Green Emotional Involvement indirectly promotes Consumer Engagement Behavior by enhancing Perceived Authenticity.

H3: Perceived Authenticity plays a significant mediating role between Green Emotional Involvement and Consumer Engagement Behavior.

#### 2.2.4 The chain mediating role of Perceived Authenticity and Perceived Value

Perceived Authenticity refers to the degree of trust consumers place in the environmental attributes and health values claimed by green products, representing their subjective judgment of the credibility of green information (Kim and Song, 2020). When consumers believe that the environmental claims of a green product are truthful and credible, their perception of the product’s functional benefits, emotional benefits, and altruistic value is significantly enhanced, thereby increasing the overall Perceived Value (Sun et al., 2022). Studies have shown that Perceived Authenticity strengthens consumers’ positive perceptions of green products by reducing information asymmetry and uncertainty, which in turn improves Perceived Value (Elshaer et al., 2025).

Green Emotional Involvement has a significant positive effect on Perceived Authenticity. Consumers with high Green Emotional Involvement pay closer attention to the environmental attributes of green products and actively seek to verify green information, thereby increasing trust in green claims (Atkinson and Rosenthal, 2014). Perceived Authenticity further enhances Perceived Value, leading consumers to assign higher evaluations to the functional, emotional, and social values of green products (Yang et al., 2023). Ultimately, high Perceived Value drives consumers to establish a deeper alignment with the brand, reflected in stronger purchase intentions, repeat purchase behaviors, and brand interactions (Naseem et al., 2015).

In this chain mediation model, Green Emotional Involvement first indirectly enhances Perceived Value by increasing Perceived Authenticity; subsequently, Perceived Value promotes Consumer Engagement Behavior. Just as an interesting study shows that user engagement with ubiquitous computing devices hinges on emotional resonance and self-congruity to drive adoption or loyalty, green consumer engagement is similarly fueled by Green Emotional Involvement (Hussain et al., 2019). This transmission pathway reveals how Green Emotional Involvement influences Consumer Engagement Behavior through the dual mediating effects of Perceived Authenticity and Perceived Value.

H4: Perceived Authenticity and Perceived Value play a chain mediating role between Green Emotional Involvement and Consumer Engagement Behavior.

**FIGURE 1:**
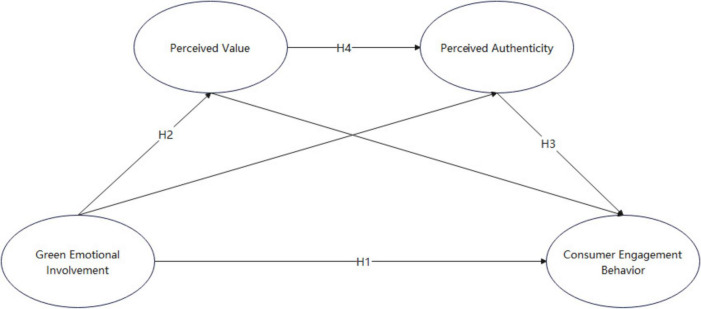
Research hypothesized model of the impact of Green Emotional Involvement on Consumer Engagement Behavior. GEI, Green Emotional Involvement; PV, Perceived Value; PA, Perceived Authenticity; CEB, Consumer Engagement Behavior.

Based on the above hypotheses, the conceptual model for this study is presented in Figure 1.

## 3 Research design and methodology

### 3.1 Research design

This study employed a quantitative, cross-sectional survey design to examine the mechanism by which Green Emotional Involvement (GEI) influences Consumer Engagement Behavior (CEB). Based on the Theory of Planned Behavior (TPB), a conceptual model was developed that integrates two mediating variables—Perceived Value (PV) and Perceived Authenticity (PA)—and a moderating variable (gender).

### 3.2 Measurement instruments

All variables were measured using established scales adapted from prior research to ensure validity and reliability. Items were rated on a five-point Likert scale ranging from 1 (“Strongly disagree”) to 5 (“Strongly agree”).

Green Emotional Involvement (GEI): adapted from Guo et al. (2025), comprising three items reflecting consumers’ sensitivity and emotional attention to green product information.

Perceived Value (PV): adapted from Riva et al. (2022), Saut and Bie (2024), including six items capturing trust and credibility in green product claims.

Perceived Authenticity (PA): adapted from Loebnitz et al. (2022), Sun et al. (2022), including six items capturing trust and credibility in green product claims.

Consumer Engagement Behavior (CEB): derived from Van Doorn et al. (2010), Hao (2017), Yan (2023), including five items reflecting proactive sharing, participation, and recommendation behaviors.

### 3.3 Data collection and sample

The formal survey was administered between July 1 and August 15, 2025, via the professional online platform Wenjuanxing^1^. A combination of purposive and maximum variation sampling ensured representation across age, gender, and occupation. Of 300 distributed questionnaires, 274 valid responses were obtained after excluding incomplete or patterned responses.

The sample comprised 50.2% male and 49.8% female participants; most respondents held a bachelor’s degree (50.9%) and were employed in enterprises (39.9%). This distribution indicates a balanced and representative respondent structure.

### 3.4 Data analysis procedures

Data were analyzed using SPSS 26.0 and AMOS 24.0, the analytical process included:

1. Descriptive statistics to profile respondents;
2. Reliability and validity testing (Cronbach’s α, AVE, CR);
3. Confirmatory Factor Analysis (CFA) to assess measurement model fit;
4. Structural Equation Modeling (SEM) to test hypothesized relationships;
5. Bootstrapping (2,000 resamples) to verify mediating effects;
6. Multi-group analysis (MGA) to examine gender moderation effects.

These procedures ensured methodological rigor and robustness of findings.

## 4 Research results

### 4.1 Descriptive statistical analysis

The questionnaire consisted of two parts: demographic information and 24 test items. As shown in Table 1, the sample comprised 50.2% male respondents and 49.8% female respondents. In terms of educational background, 7.3% had a junior high school education or below, 15.8% had completed high school or technical secondary school, 16.8% held a junior college degree, 50.9% held a bachelor’s degree, and 9.2% had a master’s degree or above.

**TABLE 1:** Demographic information.

Category	Option	Frequency	Percentage (%)
Gender	Male	137	50.2
	Female	136	49.8
Education	Junior high school and below	20	7.3
	High school/vocational school	43	15.8
	College degree	46	16.8
	Bachelor degree	139	50.9
	Graduate students and above	25	9.2
Career	Enterprise staff	109	39.9
	Institution staff	54	19.8
	Government officials	32	11.7
	Students	44	16.1
	Free staff	16	5.9
	Others	18	6.6
Monthly income level	3,000 and below	58	21.2
	3,001–5,000	68	24.9
	5,001–8,000	71	26.0
	8,001–12,000	43	15.8
	12,000 and above	33	12.1

Regarding occupational distribution, 39.9% were enterprise employees, 19.8% were employees in public institutions, 11.7% worked in government departments, 16.1% were students, 5.9% were self-employed, and 6.6% were in other occupations.

Monthly income levels were distributed as follows: 21.2% earned 3,000 RMB or below, 24.9% earned between 3,001 and 5,000 RMB, 26.0% earned between 5,001 and 8,000 RMB, 15.8% earned between 8,001 and 12,000 RMB, and 12.1% earned 12,000 RMB or above. The demographic characteristics of the sample indicate a reasonable distribution, providing a reliable basis for subsequent research inferences.

### 4.2 Common method bias test

To examine whether the data were affected by serious common method bias, this study employed Harman’s (1976) single-factor test for statistical analysis. Specifically, all measurement items for the variables (a total of 23 items) were subjected to principal component factor analysis, using an unrotated principal component extraction method.

The results showed that six factors with eigenvalues greater than 1 were extracted, accounting for a cumulative variance of 77.294%. The first factor explained 18.666% of the variance, which is well below the critical threshold of 50% (Podsakoff et al., 2003). This indicates that no single factor accounted for the majority of the variance, suggesting that the data were not substantially affected by common method bias and met the prerequisites for structural equation modeling analysis.

### 4.3 Reliability and validity tests

This study used SPSS 26.0 and AMOS 24.0 to examine the reliability and validity of the measurement scales. The results are shown in Table 2.

**TABLE 2:** Reliability and convergent validity testing.

Dimension	Items	Unstd.	S.E.	*Z*	*P*	Std.	Cronbach’s α	CR	AVE
GEI	GEI1	1.067	0.083	12.898	***	0.766	0.892	0.892	0.762
	GEI2	1.017	0.077	13.217	***	0.782			
	GEI3	1.099	0.081	13.626	***	0.804			
	GEI4	0.964	0.078	12.427	***	0.741			
	GEI5	0.969	0.081	11.916	***	0.714			
	GEI6	1.000	–	–	–	0.762			
PV	PV1	1.000	–	–	–	0.783	0.874	0.874	0.582
	PV2	0.995	0.078	12.783	***	0.752			
	PV3	0.921	0.076	12.110	***	0.717			
	PV4	1.041	0.077	13.492	***	0.787			
	PV5	0.988	0.075	13.231	***	0.774			
PA	PA1	1.000	–	–	–	0.773	0.886	0.886	0.608
	PA2	1.012	0.076	13.355	***	0.785			
	PA3	1.036	0.079	13.080	***	0.771			
	PA4	1.035	0.076	13.603	***	0.797			
	PA5	0.986	0.075	13.143	***	0.774			
CEB	CEB1	0.911	0.074	12.344	***	0.712	0.875	0.876	0.586
	CEB2	0.960	0.068	14.073	***	0.792			
	CEB3	0.951	0.075	12.733	***	0.730			
	CEB4	1.010	0.073	13.887	***	0.784			
	CEB5	1.000	–	–	–	0.806			

***p < 0.001.

In terms of reliability, the Cronbach’s α coefficients for the four latent variables—Green Emotional Involvement(GEI), Perceived Value (PV), Perceived Authenticity (PA), and Consumer Engagement Behavior(CEB)—were 0.892, 0.886, 0.875, and 0.876, respectively, all exceeding the recommended threshold of 0.80. This indicates that the scales possess high internal consistency. In addition, the Cronbach’s α values for all measurement items did not show any significant increase, suggesting that all items have retention value.

In terms of validity testing, a confirmatory factor analysis (CFA) was conducted. The standardized factor loadings for all items ranged from 0.614 to 0.844, all significantly greater than 0.60, indicating a good level of convergence between the measurement indicators and their respective constructs. Further, the composite reliability (CR) and average variance extracted (AVE) for each construct were calculated, with all CR values exceeding 0.80 and all AVE values above 0.560, meeting the criteria for convergent validity.

To further verify discriminant validity, the Fornell and Larcker (1981) criterion was applied to analyze the correlations among the variables. The results showed that the square roots of AVE for all variables (GEI = 0.762, PV = 0.763, PA = 0.780, CEB = 0.766) were greater than the highest correlation coefficients between each variable and any other construct (maximum correlation = 0.601). This indicates that the constructs used in this study have good discriminant validity.

In conclusion, the measurement indicators of all latent variables in this study demonstrated strong reliability, convergent validity, and discriminant validity, providing a solid data foundation for subsequent structural equation modeling analysis.

### 4.4 Model fit test

AMOS 24.0 was used to assess the goodness-of-fit of the structural equation model. The fit indices considered included the chi-square to degrees of freedom ratio (χ^2^/df), absolute fit indices (e.g., GFI, AGFI, TLI, CFI, IFI, NFI, NNFI), and incremental fit indices (e.g., RMSEA, SRMR, RMR). The criteria for evaluation were as follows: χ^2^/df less than 3 indicates good model fit; GFI, AGFI, TLI, CFI, IFI, NFI, and NNFI values greater than 0.900 and closer to 1 suggest a high degree of model-data fit; RMSEA less than 0.08, SRMR less than 0.08, and RMR less than 0.05 indicate low model error and desirable fit.

The analysis results were as follows (revealed in Table 3): χ^2^/df = 1.702, GFI = 0.907, AGFI = 0.883, TLI = 0.955, CFI = 0.961, IFI = 0.961, NFI = 0.911, NNFI = 0.955, RMSEA = 0.051, SRMR = 0.041, RMR = 0.054, and the RMSEA 90% confidence interval was 0.041–0.060. All values met the standard requirements for structural equation model fit (e.g., χ^2^/df < 2 indicates excellent fit; GFI ≥ 0.90 is acceptable; AGFI values closer to 1 indicate better fit; TLI ≥ 0.90 generally reflects excellent fit; RMSEA values closer to 0 suggest better fit; NFI ≥ 0.90, TLI ≥ 0.90, and CFI ≥ 0.90 are indicative of very good fit).

**TABLE 3:** Model fitting indicators.

Common indicators	χ 2	*df*	*P*	χ 2/*df*	GFI	RMSEA	RMR	CFI	NFI	NNFI
Criteria	–	–	>0.05	<3	>0.9	<0.10	<0.05	>0.9	>0.9	>0.9
Value	311.438	183	0	1.702	0.907	0.051	0.054	0.961	0.911	0.955
Other indicators	TLI	AGFI	IFI	PGFI	PNFI	PCFI	SRMR	–	–	–
Criteria	>0.9	>0.9	>0.9	>0.5	>0.5	>0.5	<0.1	–	–	–
Value	0.955	0.883	0.961	0.719	0.794	0.837	0.041	–	–	–

When the default model is set,χ^2^(210) = 3507.893, *p* = 1.000, AIC = 93.718, BIC = 266.973.

Therefore, the structural model developed in this study demonstrates good overall fit and effectively reflects the relationship between the theoretical model and the empirical data.

### 4.5 Hypothesis testing of the model

#### 4.5.1 Direct effect test

To verify whether the direct path from Green Emotional Involvement (GEI) to Consumer Engagement Behavior (CEB) is significant, this study constructed a structural equation path model using hierarchical regression analysis to test the direct effect relationships among the main variables.

The analysis results show that GEI has a significant positive effect on CEB (β = 0.429, *t* = 6.552, *p* < 0.001), indicating that the higher the degree of consumers’ emotional involvement in green concepts, the stronger their engagement behavior at the brand or enterprise level. Therefore, Hypothesis H1 is supported.

In addition, GEI exhibits significant positive effects on both Perceived Value (PV) and Perceived Authenticity (PA) (β = 0.519, *p* < 0.001; β = 0.377, *p* < 0.001), suggesting that higher emotional involvement increases consumers’ subjective value assessments and perceptions of authenticity regarding green products or brands. The results also show that PV (β = 0.279, *p* < 0.001) has a significant positive effect on CEB, whereas PA (β = 0.081, *p* > 0.05) does not have a statistically significant direct effect on CEB.

Notably, the use of CB-SEM in this study allowed for a nuanced examination of the structural relationships, as this method is particularly effective in disentangling the subtle, multilayered interplay among a multitude of variables (Anwar et al., 2025), thereby enhancing the reliability of the observed effects.

In summary, the direct path from GEI to CEB is significant, and GEI exerts positive effects on both mediating variables (PV and PA). Among them, PV also has a significant direct effect on CEB, providing preliminary support for the subsequent mediation effect analysis.

#### 4.5.2 Mediation effect test

To further explore the mediating pathways through which Green Emotional Involvement (GEI) influence Consumer Engagement Behavior (CEB) via Perceived Value (PV) and Perceived Authenticity (PA), this study employed multiple regression analysis combined with the bootstrap method to test the chain mediation effect. Following MacKinnon et al. (2007) the bias-corrected approach in the nonparametric bootstrap method was adopted, as it has been experimentally shown to be optimal. Therefore, only the bias-corrected confidence intervals are reported.

The mediation analysis results (illustrated in Table 4) indicate that the total effect of GEI on CEB is 0.632. The mediating effect of PV is significant (confidence interval does not include zero, *p* < 0.05), whereas the mediating effect of PA is not statistically significant.

**TABLE 4:** Summary of the effect analysis process.

Effect	Title	Effect	*SE*	*t*	*P*	LLCI	ULCI
Direct effect	GEI⇒CEB	0.429	0.065	6.552	0	0.3	0.557
Indirect effect process	GEI⇒PV	0.538	0.052	10.246	0	0.435	0.641
	GEI⇒PA	0.377	0.056	6.713	0	0.267	0.487
	PV⇒PA	0.371	0.056	6.68	0	0.262	0.48
	PV⇒CEB	0.288	0.065	4.455	0	0.161	0.414
	PA⇒CEB	0.085	0.066	1.282	0.201	−0.045	0.214
Total effect	GEI⇒CEB	0.632	0.054	11.73	0	0.526	0.738

LLCI refers to the lower 95% range of the estimate and ULCI refers to the upper limit of the 95% range of the estimate.

To further confirm the significance of the mediation effects, this study applied the bootstrap method (with 2,000 resamples) to test the significance of the indirect effects. The results are shown in Table 5 below:

**TABLE 5:** Mediation effect analysis.

Title	Effect	Boot SE	BootLLCI	BootULCI	*z*	*P*
GEI⇒PV⇒CEB	0.155	0.046	0.051	0.232	3.374	0.001
GEI⇒PA⇒CEB	0.032	0.034	−0.038	0.1	0.935	0.35
GEI⇒PV⇒PA⇒CEB	0.017	0.019	−0.019	0.056	0.895	0.371

BootLLCI refers to the lower limit of the 95% interval of Bootstrap sampling, BootULCI refers to the upper limit of the 95% interval of Bootstrap sampling, and the bootstrap type: percentile bootstrap method. The gray shading is the chain mediator, and the rest is the parallel intermediary.

The indirect effect of GEI→PV→CEB was 0.155, with a 95% bootstrap confidence interval of [0.051, 0.232], which does not include zero, indicating that the pathway with Perceived Value as a mediator is significant. The indirect effect of GEI→PA→CEB was 0.032, with a 95% confidence interval of [−0.038, 0.100], which includes zero, suggesting that the pathway with Perceived Authenticity as a mediator is not significant. The chain mediation effect of GEI→PV→PA→CEB was 0.017, with a 95% confidence interval of [−0.019, 0.056], which also includes zero, indicating that the pathway with Perceived Value and Perceived Authenticity as sequential mediators is not supported.

In summary, Perceived Value plays a significant mediating role between Green Emotional Involvement and Consumer Engagement Behavior, whereas the mediating effects of Perceived Authenticity and the chain mediation pathway did not receive statistical support. Therefore, Hypothesis H2 is supported, while H3 and H4 are not supported.

#### 4.5.3 Gender moderation effect test

To examine the moderating role of gender in the research model and further explore whether differences exist between male and female consumers in the path from Green Emotional Involvement (GEI) to Consumer Engagement Behavior (CEB), this study employed the multi-group structural equation modeling (Multi-group SEM) approach. Specifically, an unconstrained model (baseline model) and a constrained model (in which path coefficients were set equal across groups) were constructed, and chi-square difference tests (Δχ^2^) were performed to determine whether significant gender differences existed in the model structure.

First, the baseline model was established without imposing any equality constraints on the parameters. Then, equality constraints were applied to each path in turn to construct partially constrained models, and the chi-square difference between each constrained model and the baseline model was compared. The analysis results are shown in Table 6.

**TABLE 6:** Gender moderating effect test.

Model	Chi-square (Δ c^2^)	*P*	Degrees of freedom difference (Δ df)
1. Basic path	–	–	–
2. Constrained path GEI→PV	0.222	0.638	1
3. Constrained path PV→CEB	0.415	0.519	1
4. Constrained path GEI→PA	4.487	0.034**	1
5. Constrained path PV→PA	0.343	0.558	1
6. Constrained path PA→CEB	0.733	0.392	1
7. Constrained path GEI→CEB	1.425	0.233	1

**p < 0.01.

For the constrained path Green Emotional Involvement→Perceived Authenticity, the chi-square difference was Δχ^2^ = 4.487 with *p* = 0.034 < 0.05, reaching a statistically significant level. This indicates that this path differs significantly between gender groups.

In contrast, for the other paths Green Emotional Involvement→Perceived Value, Perceived Value→Consumer Engagement Behavior, and Perceived Authenticity→Consumer Engagement Behavior, the Δχ^2^ values did not reach statistical significance (*p* > 0.05), suggesting that there are no significant gender differences in these relationships.

A further comparison of path coefficients between groups (results in Table 7) revealed that, for female consumers, the path coefficient of Green Emotional Involvement → Perceived Authenticity was 0.506 (*t* = 3.957), whereas for male consumers it was 0.355 (*t* = 3.682). Although both coefficients were statistically significant, the effect was stronger among female consumers. This suggests that, compared to men, women are more likely to enhance their perception of brand authenticity as a result of higher Green Emotional Involvement.

**TABLE 7:** Comparison of gender moderating effects.

Path	Female consumers		Male consumers	
	Standardization coefficient	*t*	Standardization coefficient	*t*
GEI→PV	0.655***	6.156	0.616***	6.062
PV→CEB	0.206	1.607	0.449***	3.407
GEI→PA	0.235**	2.025	0.527***	5.296
PV→PA	0.506***	3.957	0.355***	3.682
PA→CEB	0.095	0.841	−0.043	−0.319
GEI→CEB	0.5***	4.193	0.449***	3.399

***Means that it is significant at the level of 1%.

**Indicates significant at the 5% level.

In addition, for the paths Green Emotional Involvement → Perceived Value, Perceived Value → Consumer Engagement Behavior, and Green Emotional Involvement → Consumer Engagement Behavior, significant relationships were observed for both genders; however, the comparison of path coefficients did not reveal statistically significant differences. This indicates that gender does not exert a significant moderating effect on these paths.

In summary, gender exhibits a significant moderating effect on the path Green Emotional Involvement → Perceived Authenticity, but not on the other paths. This finding suggests that, in promoting green emotional marketing, enterprises could strengthen strategies for shaping and conveying perceptions of authenticity specifically targeted toward female consumers, thereby eliciting stronger engagement behavior.

## 5 Discussion

### 5.1 Summary of key findings

This study set out to clarify how Green Emotional Involvement shapes consumers’ engagement with green food in the Chinese market. The main empirical results are:

First, stronger emotional attachment to environmental attributes directly increases consumers’ likelihood of interacting with, sharing, and advocating for green-food brands.

Second, Perceived Value acts as the principal mediator: emotional involvement boosts consumers’ assessments of functional, emotional, and social benefits, and this heightened value perception is the main conduit turning positive feelings into active engagement.

Third, Perceived Authenticity plays only a supporting role; although emotional involvement elevates authenticity judgments, these judgments do not exert a significant subsequent effect on engagement, so authenticity serves as a secondary cue rather than a behavioral driver.

Fourth, gender moderates the involvement–authenticity link, with female consumers displaying a significantly stronger path coefficient than males, implying that women give greater weight to credibility cues once emotionally involved.

Finally, the chain involving authenticity before value is statistically negligible, reaffirming that value perception—not authenticity verification—is the decisive step that converts green emotional attachment into visible consumer engagement.

### 5.2 Theoretical implications

This research contributes to the literature in three aspects.

First, it advances the TPB framework by integrating emotional and cognitive components, demonstrating that emotional involvement is not merely an attitudinal variable but a motivational driver of engagement.

Second, it refines the dual-mediation mechanism by identifying the differential roles of PV and PA, highlighting that affective and rational evaluations exert unequal impacts in sustainable consumption contexts.

Third, it introduces gender as a moderating factor, offering a novel perspective on demographic heterogeneity and extending prior findings on gender differences in emotional processing and sustainability communication.

### 5.3 Practical implications

From a managerial and policy perspective, this study provides several actionable insights: Enhance emotional storytelling: Green enterprises should design marketing strategies that evoke positive emotions, empathy, and environmental identification to strengthen GEI.

Increase perceived value: Emphasizing tangible benefits (e.g., product quality, eco-efficiency) and symbolic meanings (e.g., personal identity, moral satisfaction) can effectively raise PV and engagement.

Reinforce authenticity systems: Transparent labeling, third-party certification, and consistent green communication can improve consumers’ trust in environmental claims.

Develop gender-sensitive marketing: Female consumers are more responsive to emotional narratives, whereas male consumers focus on practical outcomes—thus, tailored communication enhances effectiveness.

Support public policy: Policymakers may use these findings to promote nationwide green literacy campaigns, cultivating consumers’ confidence in verified sustainable products.

### 5.4 Limitations and future research

Although the study provides valuable theoretical and empirical insights, several limitations remain.

First, the data were collected exclusively from Chinese respondents, which restricts generalizability. Future studies should employ cross-cultural comparative designs to validate whether these mechanisms hold in different socio-cultural contexts. For instance, in markets where credible eco-labels are scarce or skepticism is high, authenticity cues might lose diagnostic power, so buyers fall back on brand fame or price, widening the attitude–behavior gap the cues aimed to close.

Second, the cross-sectional design limits causal inference. Longitudinal or experimental studies could better capture the dynamic evolution of emotional and cognitive mechanisms.

Third, the non-significant mediating effect of PA indicates that authenticity perception may be context-specific. Future research could explore multi-dimensional authenticity (functional, moral, symbolic) and its interaction with consumer experience or brand reputation.

Fourth, other potential moderators—such as environmental identity, trust, or cultural values—could be integrated to enrich the model’s explanatory power.

## 6 Conclusion

Grounded in the Theory of Planned Behavior and Involvement Theory, this study systematically explored the influence mechanism of Green Emotional Involvement (GEI) on Consumer Engagement Behavior (CEB). It did so by integrating Perceived Value (PV) and Perceived Authenticity (PA) as mediating variables. Gender was included as a moderating variable.

All analyses were conducted within the Theory of Planned Behavior (TPB) framework. The results show that GEI significantly and positively affects CEB, confirming that consumers’ emotional attachment to green products promotes their active participation, recommendation, and interaction behaviors. PV plays a significant mediating role, indicating that consumers’ perceived benefits and satisfaction are critical drivers of engagement. However, PA does not show a significant mediating effect, implying that authenticity perceptions may be less salient. This occurs in contexts where consumers have limited exposure to reliable green certifications.

It also happens when consumers lack trust in environmental claims. Gender moderates the relationship between GEI and PA: female consumers exhibit higher sensitivity to authenticity cues, while male consumers respond more to value-based evaluations. These results enrich the theoretical understanding of gender heterogeneity in green consumer psychology, also provide empirical support for the differentiated influence of affective and cognitive mechanisms.

Overall, the study confirms the asymmetric effect of emotional and cognitive paths in shaping green engagement behavior and extends TPB by incorporating emotional involvement as a key antecedent variable.

## Data Availability

The original contributions presented in this study are included in this article/supplementary material, further inquiries can be directed to the corresponding author.

## Footnotes

1
http://www.wjxwww.wjx
